# Pediatric Anesthesia: Excellence in Patient Care

**DOI:** 10.3390/jcm15114073

**Published:** 2026-05-25

**Authors:** Peter Marhofer, Markus Zadrazil, Philipp Opfermann

**Affiliations:** Department of Anesthesia, Critical Care and Pain Medicine, Medical University of Vienna, Waehringer Guertel 18–20, 1090 Vienna, Austria

Over the last 25 years, pediatric anesthesia has emerged as a sub-specialty within anesthesia and intensive care. The degree of specialization differs across countries, and debate continues over how many sub-specialties should exist within the field. Regardless, it seems obvious that particular patient populations require particularly educated medical specialists. Children represent a sensitive patient population, with hemodynamic, respiratory, metabolic, pharmacological and anatomical characteristics requiring appropriate management. Anesthesia is integral to the perioperative care of pediatric patients, encompassing preoperative assessment to postoperative pain management.

The present editorial refers to a Special Issue in the field of pediatric anesthesia, where several topics, from pre- to postoperative management, as well as basic research topics are discussed. Al Mutair et al. investigate in a meta-analysis the impact of intranasal dexmedetomidine on emerge delirium and agitation in children undergoing ENT surgery [1]. The authors conclude that the intranasal administration of dexmedetomidine, a potent alpha-2-receptor agonist, may reduce the occurrence of delirium and agitation in pediatric anesthesia, but more research is needed to validate its clinical impact and to define dosing regimens. The intranasal administration of sedative drugs was also investigated in a second systematic review and meta-analysis by Haridoss et al., where dexmedetomidine, midazolam and ketamine were used in pediatric dentistry [2]. The authors of this study found that intranasal sedation with various drugs is safe and effective in pediatric dentistry, but they also complained about the absence of standardized sedation protocols. It is worth mentioning that Haridoss et al. describe concerns regarding the overall bias judgement (from “some concerns” to “high risk concerns”) in 90% of included studies, thus illustrating the challenge in systematic reviews and meta-analyses. Heterogeneity and the overall quality of underlying publications are described as the main concerns of systematic reviews and meta-analyses [3]. The prehospital assessment for pediatric patients with thermal injuries, with a particular focus on fluid therapy, is discussed by Frank et al. [4]. In a retrospective analysis of 104 patients, they found a significant overestimation of the burned total body surface area, with no influence on fluid therapy decisions. Interestingly, pain therapy showed gender differences, where girls received less analgesics as compared with boys. The impact of congenital cyanotic and acyanotic cardiac surgery on the endothelial glycocalyx, which plays a fundamental role in the maintenance of plasma and vessel-wall homeostasis, is investigated by a prospective study from Schiefer et al. [5]. The authors observed significant increases in syndecan-1 levels, an endothelial biomarker, after cyanotic and acyanotic cardiac surgeries, thus showing perioperative glycocalyx degradation. This study serves as basic knowledge, with clinical relevance in need of investigation in subsequent studies. Finally, regional anesthesia plays an important role in this Special Issue, with Heschl et al. investigating the use of caudal catheters for major neonatal surgery without catheter-related complications [6]. Marhofer D. et al. and Zeilberger et al. show in prospective case series the performance of neonatal surgery (intestinal reconstruction and surgery of anorectal malformations) via epidural anesthesia without invasive airway management [7,8]. The latter studies illustrate the impact of regional anesthesia on avoiding airway management in our smallest patients’ population, which is still considered the main reason for complications in pediatric anesthesia. It needs to be highlighted that extensive surgery without airway manipulation is also possible in prone-positioned neonates, as illustrated by Zeilberger et al. [8].

Pediatric anesthesia includes a broad spectrum of anesthesia techniques, and particular knowledge in all fields of anesthesia is the prerequisite for safe perioperative management of all age pediatric groups. Pediatric anesthesiologists treat patients within a weight range between 400 g and >100 kg, and it is therefore obvious that special hand skills are required for a successful and safe performance in the daily clinical practice. Vascular access and airway management are only two examples in this context, where extremely small vessels and rapid oxygen desaturation with the subsequent need for successful airway management and ventilation are the main challenges. Vascular access is required in the vast majority of cases, but airway management can be avoided with skillful performance of regional anesthetic techniques.

Both peripheral and central vascular accesses [9,10], as well as regional anesthetic techniques [11], are currently performed with ultrasound guidance, and recent publications illustrate lower incidences of complications as compared with landmark-based techniques [12]. Therefore, these techniques contributed significantly to improve the safety of anesthesia in our smallest patients. Well-designed preclinical and clinical studies, as published in this Special Issue, are the main prerequisites for evolution in medicine—pediatric anesthesia is no exception from this general rule. However, it is important to recognize that research involving children demands the highest legal and ethical standards, as this population requires special protection [13]. Pivotal trials in the field of pharmaceutical products for all pediatric age cohorts may serve as only one example for the complexity of this important topic [14].

As a take-home message for all esteemed readers of this Special Issue and the adjacent editorial, we take the liberty to highlight the most important issues of pediatric anesthesia, which are based not only on the available literature, but also on our personal views:

- Pediatric anesthesia mainly demands specific manual skills to address its unique challenges.
- The small dimension requires exact and focused working techniques. Only a high case load enables the acquisition of routine in daily clinical practice.
- The main focus is the pediatric airway, since anatomical and physiological factors accelerate oxygen desaturation. Developments in regional anesthesia over the last 25 years allow avoidance of airway management in a significant percentage of clinical cases. In addition, technical advancements, such as high-resolution video laryngoscopy, also contributed to less dangerous airway situations.
- With increasing success rates of ultrasound guided regional anesthesia, a mainly opioid-free perioperative pain management became possible. It needs to be highlighted that the opioid crisis also includes the pediatric population and, whenever possible, opioids should be minimized or even avoided during the perioperative period [15].
- The implementation of ultrasound in the daily clinical practice of pediatric anesthesia is currently standard practice. No central venous accesses or regional anesthetic techniques should be performed without ultrasound guidance. Point-of-care ultrasound is another important application in daily clinical practice and allows fast diagnosis and management of hypovolemia, pneumothorax, or pericardial effusion [16].
- Specialized training concepts are required to improve knowledge and skills in pediatric anesthesia, including innovative methods. Augmented reality techniques may serve as only one example in this context.
- At present, we are able to manage the intra- and immediate postoperative challenges (vascular access, airway management, pain therapy) in an efficient and safe manner. The long-term postoperative outcome (pain, postoperative nausea and vomiting, enuresis, etc.) is mainly out of our focus. Therefore, particular attention needs to be focused on long-term postoperative outcome parameters.
- Economical aspects need to be considered from anesthesiologic and surgical perspectives. Anesthesiologists should work in a maximal efficient manner using the entire spectrum of our sub-specialty regarding adequate hand skills and knowledge. Surgeons are expected to operate with a high degree of collaboration, performing all surgical procedures in accordance with established standards. For instance, routine operations such as laparoscopic cholecystectomy typically do not warrant the use of time-intensive and costly robotic surgery [17,18].

Concluding this editorial, we would like to thank all authors for their contributions and all reviewers for their time to improving the preselected manuscript. The number of submitted manuscripts exceeded our expectations, and we believe that the published manuscripts and the adjacent editorial contribute significantly to further advancing the sub-specialty of pediatric anesthesia. Our primary scientific goal should be to ensure patient safety and provide thorough support for our colleagues, which contributes to a positive and healthy workplace.
